# Rationale for using the velocity–time integral and the minute distance for assessing the stroke volume and cardiac output in point-of-care settings

**DOI:** 10.1186/s13089-020-00170-x

**Published:** 2020-04-21

**Authors:** Pablo Blanco

**Affiliations:** Intensive Care Physician, Intensive Care Unit, Clínica Cruz Azul, 2651, 60 St., 7630 Necochea, Argentina

**Keywords:** Ultrasonography, Echocardiography, Stroke volume, Cardiac output, Shock, Doppler, Point-of-care

## Abstract

**Background:**

Stroke volume (SV) and cardiac output (CO) are basic hemodynamic parameters which aid in targeting organ perfusion and oxygen delivery in critically ill patients with hemodynamic instability. While there are several methods for obtaining this data, the use of transthoracic echocardiography (TTE) is gaining acceptance among intensivists and emergency physicians. With TTE, there are several points that practitioners should consider to make estimations of the SV/CO as simplest as possible and avoid confounders.

**Main body:**

With TTE, the SV is usually obtained as the product of the left ventricular outflow tract (LVOT) cross-sectional area (CSA) by the LVOT velocity–time integral (LVOT VTI); the CO results as the product of the SV and the heart rate (HR). However, there are important drawbacks, especially when obtaining the LVOT CSA and thus the impaction in the calculated SV and CO. Given that the LVOT CSA is constant, any change in the SV and CO is highly dependent on variations in the LVOT VTI; the HR contributes to CO as well. Therefore, the LVOT VTI aids in monitoring the SV without the need to calculate the LVOT CSA; the minute distance (i.e., SV × HR) aids in monitoring the CO. This approach is useful for ongoing assessment of the CO status and the patient’s response to interventions, such as fluid challenges or inotropic stimulation. When the LVOT VTI is not accurate or cannot be obtained, the mitral valve or right ventricular outflow tract VTI can also be used in the same fashion as LVOT VTI. Besides its pivotal role in hemodynamic monitoring, the LVOT VTI has been shown to predict outcomes in selected populations, such as in patients with acute decompensated HF and pulmonary embolism, where a low LVOT VTI is associated with a worse prognosis.

**Conclusion:**

The VTI and minute distance are simple, feasible and reproducible measurements to serially track the SV and CO and thus their high value in the hemodynamic monitoring of critically ill patients in point-of-care settings. In addition, the LVOT VTI is able to predict outcomes in selected populations.

## Background

In applying any hemodynamic monitoring technique, flow parameters, such as the stroke volume (SV) and cardiac output (CO), are key for targeting organ perfusion and oxygen delivery in patients with hemodynamic instability [1].

Since the SV and CO cannot be estimated reliably by clinical examination and routine assessment [2–4], several methods have been developed with the purpose to obtain these parameters in emergency and critically ill patients [2, 5]. Among them, the pulmonary artery catheter (PAC) has long been the mainstay for hemodynamic monitoring of critically ill patients; however, the usefulness of this device has been questioned with regard to its unfavorable harms/benefits ratio and thus it is nowadays abandoned in most intensive care units (ICU) around the world [5–7]. Of note, the PAC is still considered the gold standard for comparison when other SV/CO monitors are tested.

An ideal SV/CO monitor should be non-invasive, continuous or rapidly repeatable, cost effective, reproducible, reliable during various physiological states and should also have a fast response time [2]. In this regard, the latest American Society of Echocardiography guideline recommends using both TTE and/or TEE for assessment of SV and CO in determining responses to medical and surgical therapies [8]. The consensus on circulatory shock and hemodynamic monitoring by the Task Force of the European Society of Intensive Care Medicine provides a similar recommendation as well [9]. Besides its recommendation, it is important to note that there is conflicting evidence regarding the interchangeability between echocardiography and the pulmonary artery catheter or CO monitors for estimating the SV or CO, with some studies not showing a good correlation between them [10–13] and many studies showing the opposite [14–18].

TTE shows clear advantages in comparison with other methods for monitoring the SV and CO, highlighting its non-invasiveness, low costs, bedside application, avoidance of ionizing radiation, repeatability and extensive availability. In addition, TTE offers the possibility to link the SV or CO status to its causative factor, for example hypovolemia, cardiac dysfunction, cardiac tamponade, acute cor pulmonale and/or a vasodilated circulation [19]. In comparison with TTE, TEE offers equal or better diagnostic and monitoring performances; however, its invasiveness, limited availability and cost factors are major limitations for its use. Also, a standard TEE probe cannot be kept in the patient for too long [2]. In current practice, the main indication of TEE for hemodynamic monitoring is in mechanically ventilated patients who lack suitable TTE windows [2].

The focus of this article is to revisit the rationale of monitoring the SV and CO using the velocity–time integral and the minute distance as assessed by TTE, addressing their limitations, feasibility and reliability, all aspects with concrete implications to intensivists and emergency physicians.

## Calculation of the SV and CO by TTE

**Fig. 1:**
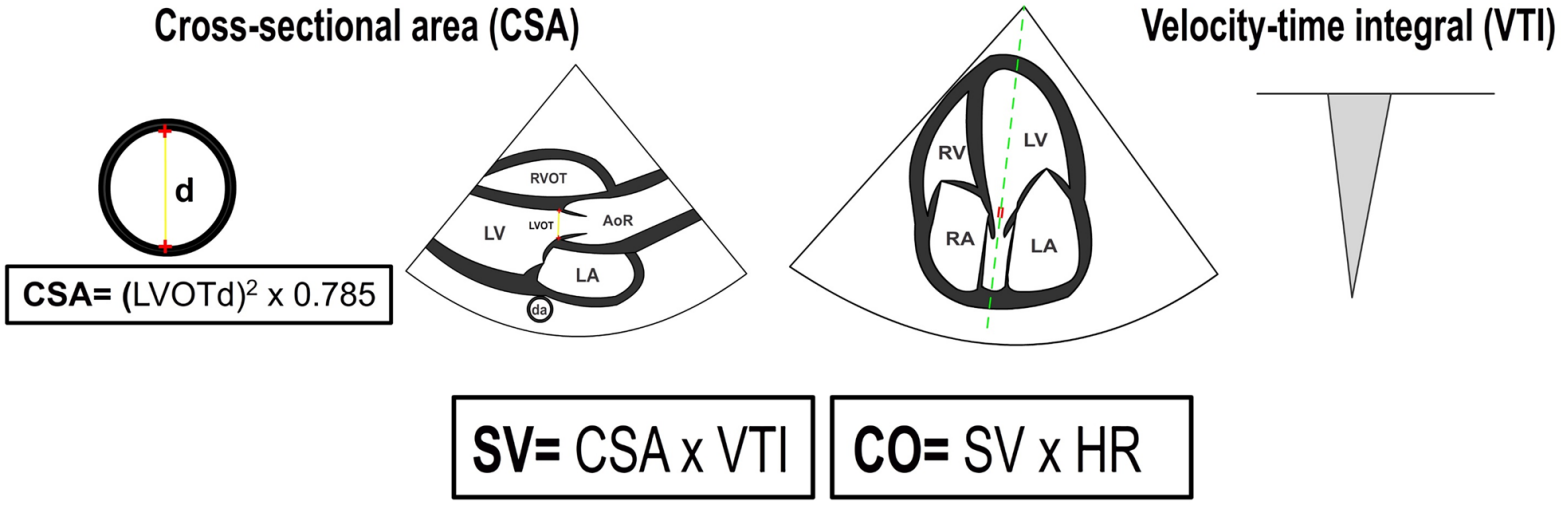
Schematic representation of the calculation of the stroke volume (SV) and cardiac output (CO) by transthoracic echocardiography. CSA: cross-sectional area; LVOT: left ventricular outflow tract; d: diameter; RVOT: right ventricular outflow tract; LV: left ventricle; AoR: aortic root; LA: left atrium; da: descending aorta; RA: right atrium; RV: right ventricle; VTI: velocity–time integral; HR: heart rate

**Fig. 2:**
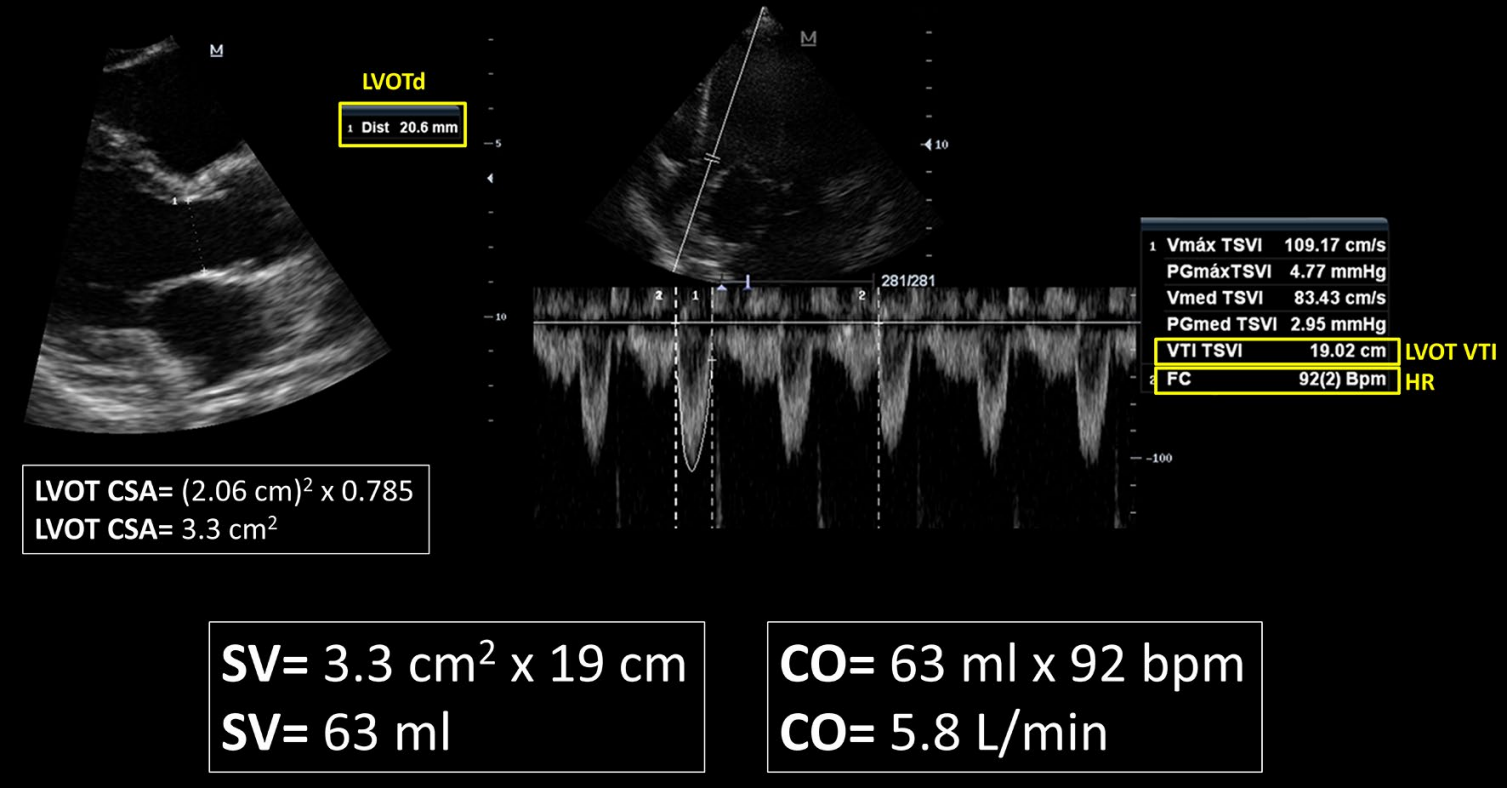
Calculation of the stroke volume (SV) and cardiac output (CO) by transthoracic echocardiography in a critically ill patient without hemodynamic compromise. LVOT: left ventricular outflow tract; d: diameter; CSA: cross-sectional area; VTI: velocity–time integral; HR: heart rate

With TTE, the SV is usually obtained from the product of the LVOT cross-sectional area (CSA, in cm^2^) with the LVOT velocity–time integral or VTI (also known as stroke distance, in cm) (Figs. 1 and 2). The LVOT CSA is derived from the LVOT diameter (LVOTd) using the formula *πr*^2^ [3.1416 × (LVOTd/2)^2^], or its equivalent (LVOTd)^2^ × 0.785. The LVOTd is acquired from the parasternal long axis view, at a mid-systolic frame, measured from the inner edge to inner edge of the LVOT or eventually between the site of insertion of the right- and non-coronary aortic leaflets [20, 21]. The LVOT VTI is obtained by tracing the envelope of the Doppler spectrum of LVOT systolic flow from the apical five- or three-chamber view using pulsed-wave Doppler (PWD), with the sample volume placed within the LVOT, approximately at 1 cm distance to the aortic valve [20]. An optimal VTI is considered when alignment of the PWD sample volume is parallel to the subaortic flow and minimal spectral broadening is obtained [1]. The product of the SV and heart rate (HR) will yield the CO (in L/min).

**Fig. 3:**
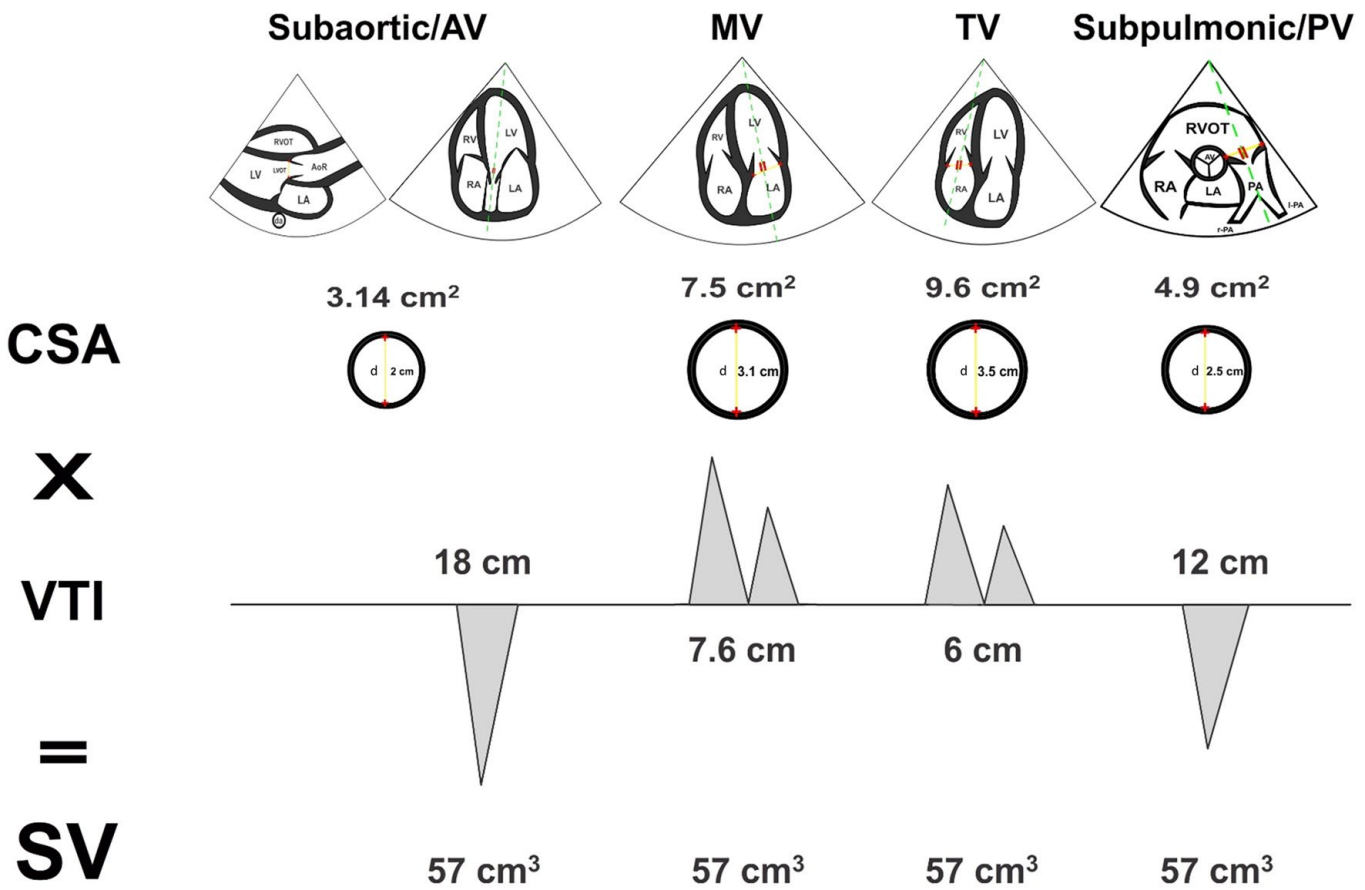
Principle of mass conservation. In the absence of valvular regurgitation or intracardiac shunts, the stroke volume (SV) is the same across each valve or orifice. CSA: cross-sectional area; VTI: velocity–time integral; AV: aortic valve; MV: mitral valve; TV: tricuspid valve; PV: pulmonary valve; LVOT: left ventricular outflow tract; d: diameter; CSA: cross-sectional area; VTI: velocity–time integral; HR: heart rate; RVOT: right ventricular outflow tract; LV: left ventricle; AoR: aortic root; LA: left atrium; da: descending aorta; RA: right atrium; RV: right ventricle; PA: pulmonary artery; r-PA: right-PA; l-PA: left-PA

Based on the principle of mass conservation, which means that the SV and CO is the same across each valve or orifice in the absence of a significant valvular regurgitation or intracardiac shunt, other sites may also be used for measurement of the SV and CO, such as the right ventricular outflow tract (RVOT) or mitral valve (MV). In these cases, the CO is equal to RVOT VTI × RVOT CSA × HR, and MV VTI × MV CSA × HR, respectively (Fig. 3) [19].

## Issues with the calculation of the SV and CO and the role of the VTI and minute distance

According to the formula used to calculate the LVOT CSA, any measurement error in the LVOTd will be squared; therefore, there might be a large error in SV calculation. Unfortunately, measurement errors are not uncommon. For example, an LVOT of 1.8 cm gives a CSA of 2.5 cm^2^, while an LVOT 2 mm higher (2 cm) gives a CSA of 3.14 cm^2^. Assuming the same LVOT VTI for each CSA (e.g., 18 cm), the SV in the first case is 45 mL, while for the second case is 57 mL (26% higher than the former). As is noted, this large difference in calculation of the SV occurs with just a minimal difference in the measurement of the LVOTd.

**Fig. 4:**
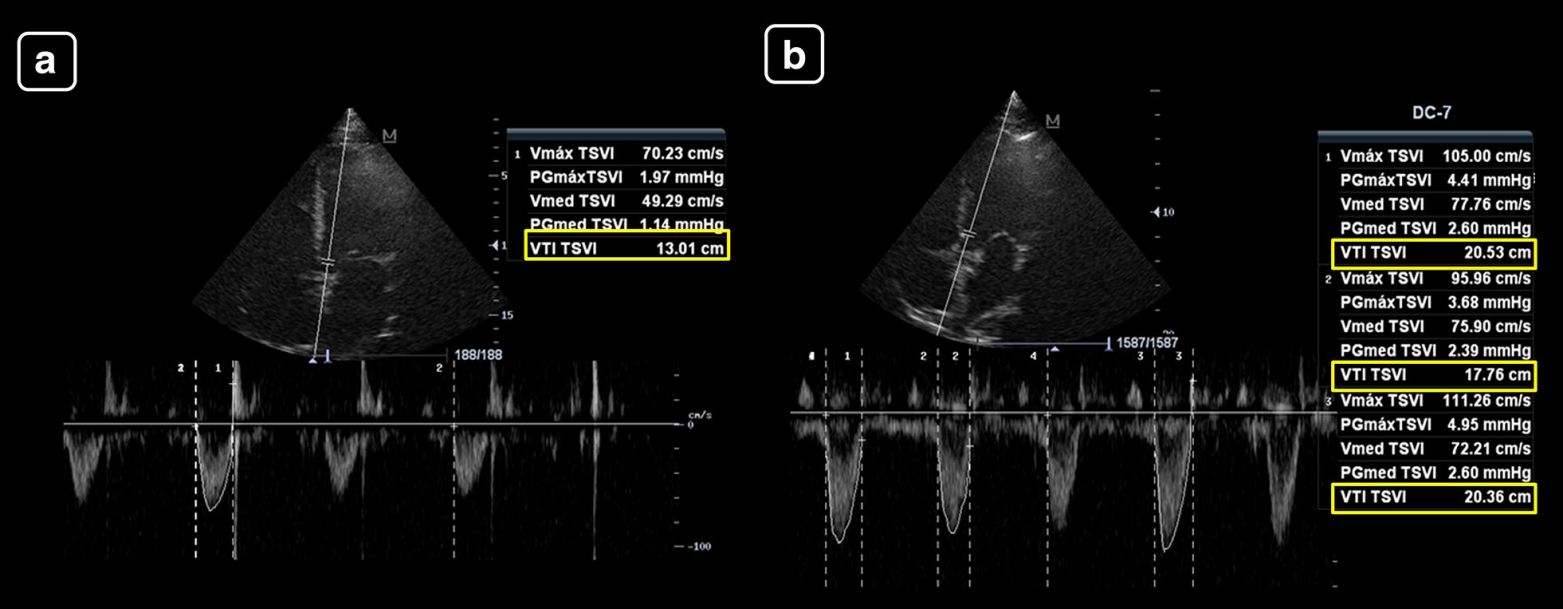
Left ventricular tract velocity–time integral (LVOT VTI, yellow boxes) for assessing changes in the SV with treatments, in a patient with suspicion of hypovolemia. **a** Baseline. **b** After a fluid challenge. A > 15% increase in the VTI indicates a positive response to therapy, as seen in this case

Given that the LVOTd is essentially constant (as is the LVOT CSA), there is no need to measure it repeatedly, and this should be done once at baseline and then the same LVOT CSA is used for serial estimations of the SV and CO. When obtaining the LVOTd is not feasible, this can also be estimated based on a published equation [0.01 × body height (cm) + 0.25] [22]. However, in practice, the LVOT CSA is rarely taken into account for tracking the SV or CO, since as is noted before, as the CSA is constant, any change in the SV must be the result of changes in LVOT VTI [21, 23, 24], while any change in the CO is due essentially to variations in the LVOT VTI, and also in the HR. Avoiding the CSA in the CO formula, the use of the VTI aids in assessing for serial changes in the SV while the minute distance, calculated as the VTI × HR, is useful to assess for serial changes in the CO (the latter is particularly useful when the HR varies significantly compared with the previous LVOT VTI measurement). Putting these concepts in practical examples:

Case 1: Patient with sepsis in whom fluid responsiveness is assessed (Fig. 4):

- Baseline measurements:
  - LVOTd = 2 cm (CSA = 3.14 cm^2^)
  - LVOT VTI = 13 cm
  - SV = 41 mL
  - HR = 80 beats/min
  - CO = 3.3 L/min
  - Minute distance = 1040 cm/min
- Measurements after a mini-fluid challenge (200 mL of crystalloids):
  - LVOTd and CSA = remain constant, equal to 3.14 cm^2^
  - LVOT VTI = 20 cm on average (increased 54% compared to baseline)
  - SV = 63 mL (increased 54% compared to baseline)
  - HR = 80 beats/min
  - CO = 5.1 L/min (increased 54% compared to baseline)
  - Minute distance = 1600 cm/min (increased 54% compared to baseline)

**Fig. 5:**
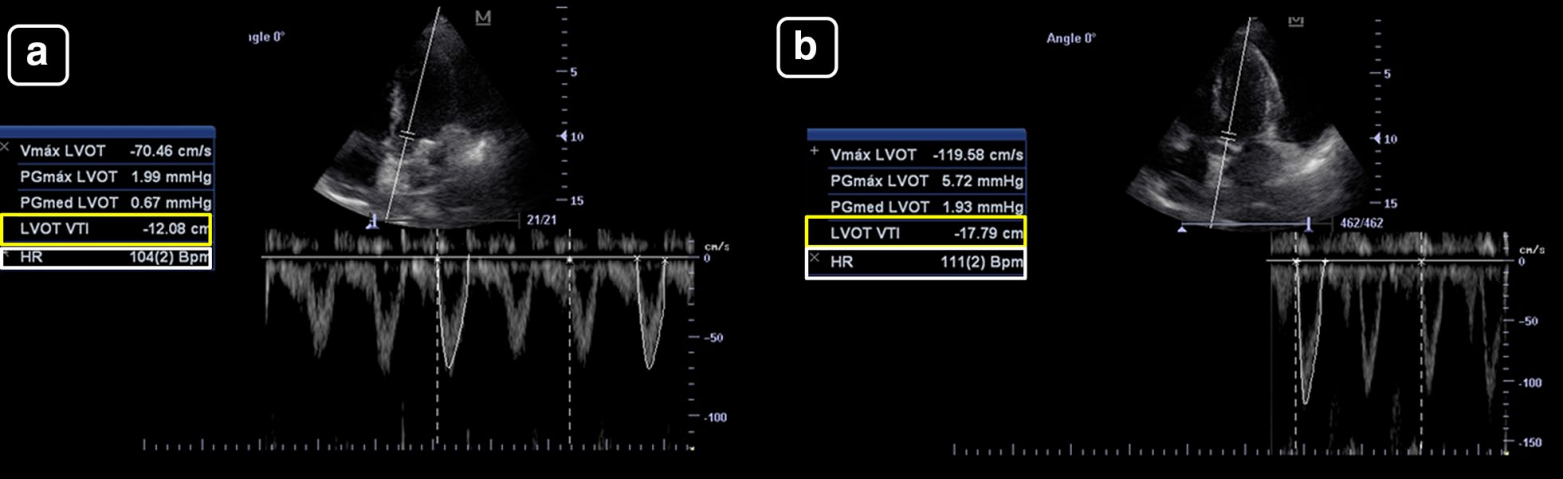
Left ventricular tract velocity–time integral (LVOT VTI, yellow boxes) and minute distance (LVOT × heart rate (HR, white boxes)) for assessing the stroke volume (SV) and cardiac output (CO) and their changes with treatments in a patient with cardiogenic shock. **a** Before treatment. **b** Receiving 10 ucg/kg/min of dobutamine. There is a marked increase in the LVOT VTI and minute distance after treatment, indicating an improvement in the SV and CO

Case 2: Patient with cardiogenic shock (Fig. 5):

- Baseline measurements:
  - LVOTd = cannot be obtained given that the PLAX was of inadequate quality
  - LVOT VTI = 12.1 cm
  - SV = –
  - HR = 104 beats/min
  - CO = –
  - Minute distance = 1248 cm/min
- Measurements on 10 ucg/kg/min of dobutamine:
  - LVOT d and CSA = remain constant, irrespective of its value
  - LVOT VTI = 17.8 cm (increased 47% compared to baseline)
  - SV = –
  - HR = 111 beats/min
  - CO = –
  - Minute distance = 1976 cm/min (increased 58% compared to baseline)

As is shown, in the first case, the main datum indicating a change in the CO is the LVOT VTI, given that the LVOT CSA and the HR remain constant; in the second case, the LVOT VTI indicates a clear improvement in the SV (47% compared to baseline) and the minute distance shows also a clear improvement in the CO (nearly 60% compared to baseline).

**Fig. 6:**
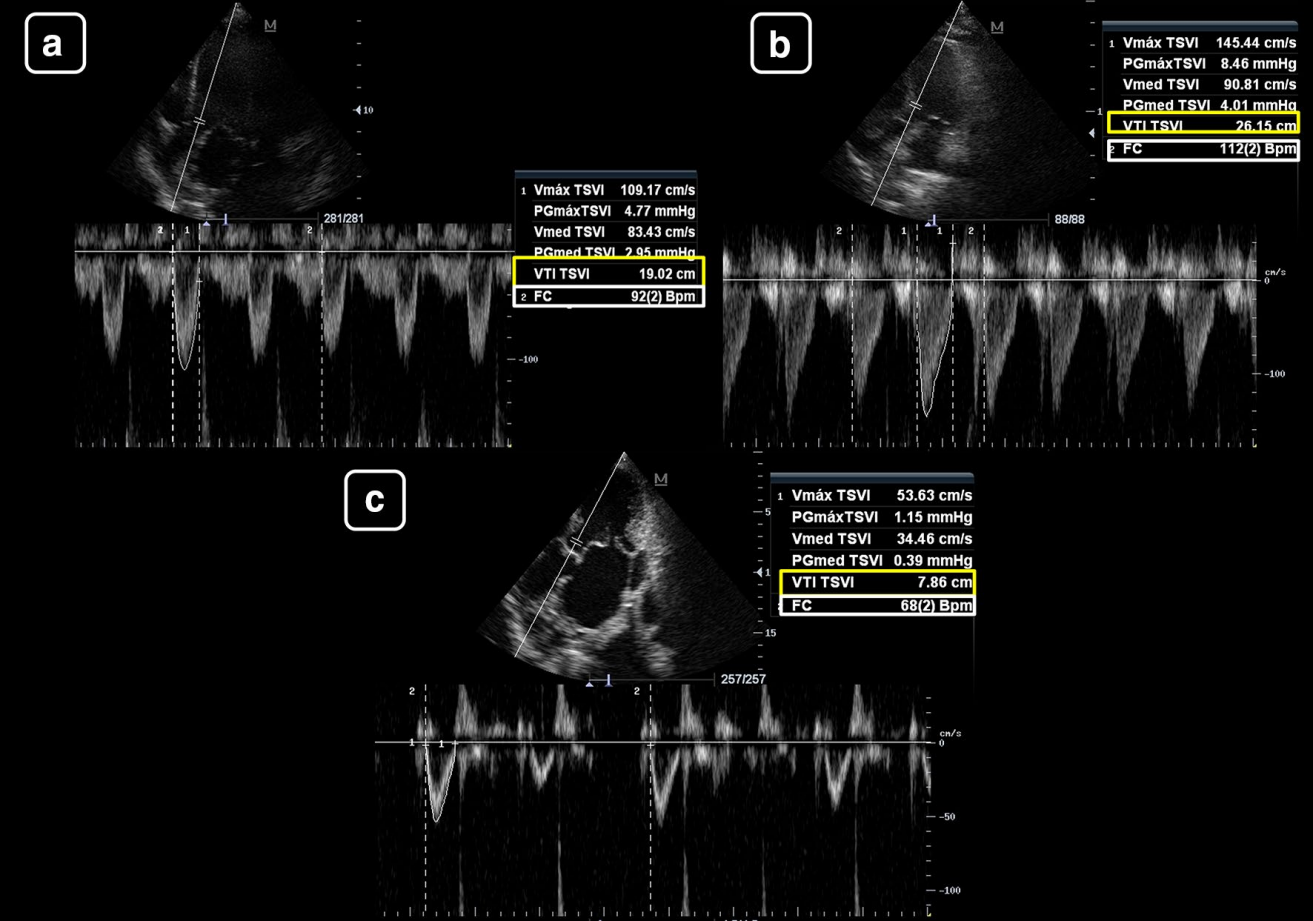
Absolute values of the left ventricular tract velocity–time integral (LVOT VTI, yellow boxes) and minute distance (LVOT × heart rate (HR, white boxes)) for estimating the stroke volume (SV) and cardiac output (CO), respectively. **a** LVOT VTI of 19 cm and HR of 93 beats/min, indicating a normal SV and CO (the minute distance is 1748 cm/min). **b** LVOT VTI of 26 cm and HR of 113 beats/min, indicating a high SV and CO (the minute distance is 2912 cm/min). **c** LVOT VTI of 8 cm and HR of 68 beats/min, indicating a low SV and CO (the minute distance is 544 cm/min)

As absolute values, Goldman et al. [25] showed that when the HR is within the normal range, mean LVOT VTI values are about 20 ± 3 cm (17–23 cm); this indicates a normal SV and CO. When the HR is under 55 bpm, the LVOT VTI values must be higher than 18 cm; otherwise, a low SV and CO are indicated and when the HR is higher than 95 bpm, LVOT VTI values must be lower than 22 cm; otherwise, a high SV and CO are suggested (Fig. 6). However, more important than using isolated values of LVOT VTI or minute distance, changes of these parameters in response to treatments are of paramount interest. This concept is the basis of functional hemodynamic monitoring, which denotes that changes in cardiac function in response to treatments are more important than single static measurements [26].

## Practical application of the VTI and the minute distance

The LVOT VTI has been used with success in several studies as a parameter for assessment of treatment responses, especially in the evaluation of fluid responsiveness [24, 26–29]. Mitral valve VTI was also used successfully for estimating volume responsiveness after a passive leg raising (PLR) test in one study [30]. From a rational point of view, the LVOT VTI (or other VTI) and the minute distance can be used to track changes in the SV and CO during the patient’s follow-up and to assess for the patient’s response to the administered treatments, like fluid challenges, vasopressor therapy, inotropic support or relief of obstructive shock mechanisms. As a rule of thumb, an increase > 15% in the VTI after a treatment indicates a concrete response to this therapy or, to the contrary, its futility in case the VTI does not change accordingly [19]. With the PLR test, a > 12% increase in the VTI after a PLR has demonstrated a good accuracy for differentiating fluid responders from non-responders [27, 28]. Besides its value in hemodynamic monitoring, there is also a role of the LVOT VTI for predicting outcomes in selected populations, as seen in patients with acute decompensated heart failure [31] and in patients with acute pulmonary embolism [32], where a very low LVOT VTI (< 10 cm) and a low LVOT VTI (< 15 cm) is associated with worse prognosis, respectively. In other study, a low LVOT VTI (< 15 cm) correlates with a low CO syndrome in patients with acute decompensated heart failure and predicts the use of inotropes [33].

## Limitations of the LVOT VTI

**Fig. 7:**
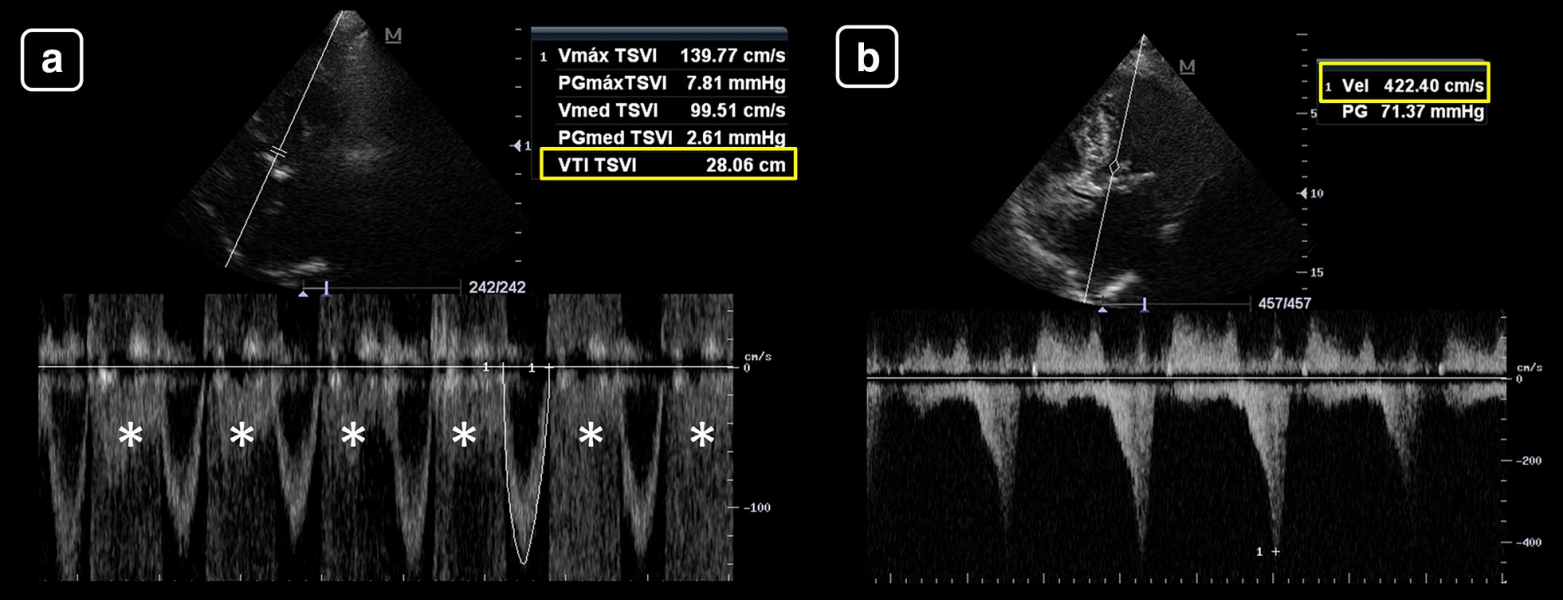
**a** Left ventricular outflow tract velocity–time integral (LVOT VTI) in severe aortic regurgitation. LVOT VTI showed high values (yellow box), overestimating the stroke volume. Asterisks: aliased spectral Doppler signal of aortic regurgitation. **b** LVOT VTI in dynamic LVOT obstruction, obtained with continuous wave Doppler (pulsed-wave Doppler waveforms were aliased). The spectral Doppler showed high blood flow peak velocities (4.2 m/s, yellow box) and a long time-to-peak signal. This situation can be seen in severe hypovolemia, hypertrophic cardiomyopathy, anterior myocardial infarctions with hypercontractile basal segments and in Takotsubo syndrome

The LVOT VTI is not reliable for estimating the SV/CO when there is a moderate-to-severe aortic regurgitation (AR) and/or a subaortic obstruction (LVOTO, fixed and/or dynamic) [19] (Fig. 7). Dynamic LVOTO may be observed in extreme hypovolemia, asymmetric left ventricular septal hypertrophy (specially at a low preload and high inotropic stimulation), anterior myocardial infarctions with compensatory hyperdynamic basal segments of the interventricular septum [19] and in Takotsubo syndrome [34]. Dynamic LVOTO and AR produce high LVOT velocities/VTI and thus overestimated VTIs. Also, for assessing the response to treatments, there is no certainty regarding whether the changes in the LVOT VTI result from an increased SV or from an increased regurgitant volume (AR) or subaortic obstruction (LVOTO).

In some patients, obtaining apical views is problematic, such as in those with lung hyperinflation and/or obese individuals.

A practical problem often observed is related to maintain the probe in the same position for measuring repeatedly the VTI with accuracy, particularly when using the PLR test. For this indication, the Probefix^®^ (i.e., an external ultrasound probe holder strapped to the patient) may have a potential value; however, in a small study using this device, there were no advantages compared with the manually recorded SV and CO using TTE [35].

As a practical issue, obtaining the LVOT VTI often requires several key strokes and may be time-consuming. As technology advances, manufacturers provide a way to do that with less effort and saving time, particularly with the utilization of the auto-VTI^®^ software. This software has been tested in an animal (piglets) experimental model of hemorrhagic shock and demonstrated a better correlation with the CO obtained by thermodilution when compared with the conventional echocardiography technique [36].

**Fig. 8:**
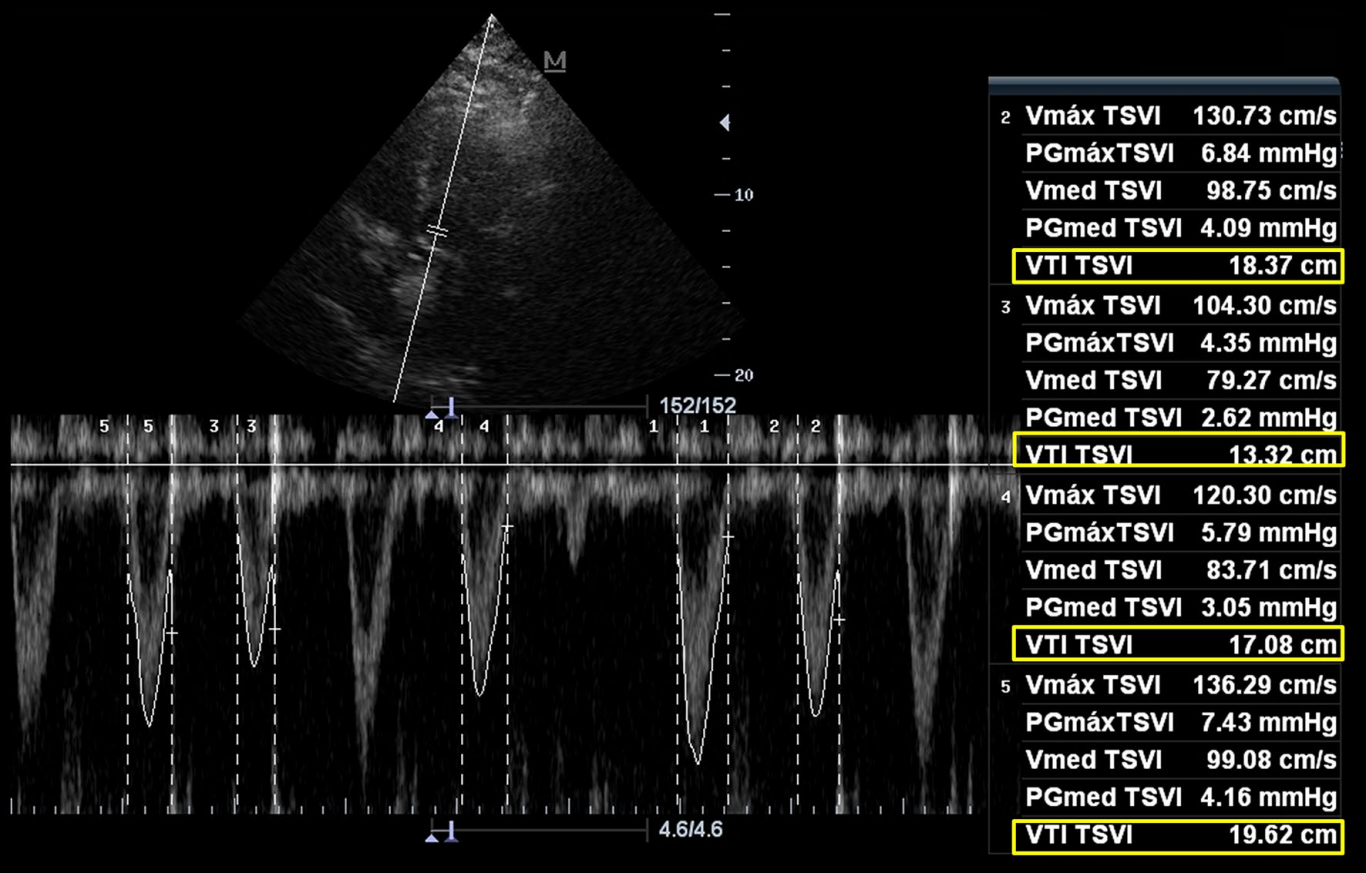
Averaging several left ventricular tract velocity–time integral (LVOT VTI, (yellow boxes)) for obtaining a mean VTI in the context of atrial fibrillation

Finally, a common issue for the accurate VTI determination is the presence of arrhythmias, especially atrial fibrillation and frequent extrasystoles, since different filling times result in beat-to-beat VTI variability. In these cases, it is recommended to average at least five VTIs in order to obtain a more accurate VTI value (Fig. 8) [23, 37].

## Feasibility and reliability for the VTI in point-of-care settings

Regarding feasibility, in the study carried out by Bergenzaun et al. [38] that evaluated echocardiographic parameters to assess the LV function in 50 patients with shock and mechanical ventilation, the LVOT VTI was obtained in 95% of all possible examinations and repeatability was high. The study carried out by Dinh et al. [1] showed that emergency physicians can accurately measure SV and CO using TTE in the emergency department. In this study including 97 patients, an optimal LVOT VTI was obtained in 78.4% of patients. More recently, McGregor et al. showed a feasibility of 78.7% for the LVOT VTI [29].

Regarding reliability, if expected physiologic responses range between increments of at least 15% in VTI after an intervention, intra- and interobserver variability for the measurement of VTI must be lower than these values, otherwise, the margin of error may exceed the patient’s physiologic response. Regarding this point, the reported intra- and interobserver variabilities are low among studies (ranging between 3 and 8%) [16, 24, 29, 38]. In a recent study, the lowest smallest change for the LVOT VTI (i.e., the smallest change that can be considered as significant and not related to the imprecision of the method or the variability of the parameter) was found to be < 5% for intra-examinations (i.e., without removing the probe from the chest wall), while it averages 11% for inter-examinations (i.e., removing the probe from the chest wall) [37].

All these data indicate that the LVOT VTI is a feasible and reliable parameter for assessing patients with hemodynamic compromise in the ICU or emergency department, particularly when measured by experienced TTE operators.

## Conclusions

Serial assessment of the SV and CO can be done measuring the VTI (LVOT, MV or RVOT) and calculating the minute distance, without the need to know the CSA. Changes in the VTI directly reflect modifications in SV while changes in the minute distance reflect modifications in the CO, and this data may aid in the ongoing assessment of the CO status and the patient’s response to treatments.

## Data Availability

Not applicable.

## Abbreviations

SV: Stroke volume
CO: Cardiac output
LVOT: Left ventricular outflow tract
VTI: Velocity–time integral
CSA: Cross-sectional area
d: Diameter
TTE: Transthoracic echocardiography
HR: Heart rate
TEE: Transesophagic echocardiography
PWD: Pulsed-wave Doppler
RVOT: Right ventricular outflow tract
MV: Mitral valve
ICU: Intensive care unit
LVOTO: Left ventricular outflow tract obstruction
PAC: Pulmonary artery catheter
PLR: Passive leg raising
